# PM_2.5_-Bound Organophosphate Esters and Childhood Sleep Disorders: Evidence from the Pearl River Delta Study

**DOI:** 10.3390/toxics14020134

**Published:** 2026-01-29

**Authors:** Li-Ping Wang, Jun Huang, Yi-Wei Wang, Jiaxiang Dong, Yun-Ting Zhang, Wen-Wen Bao, Yang Zhou, Jing-Wen Huang, Li-Xia Liang, Muhammad Amjad, Pei-Pei Wang

**Affiliations:** 1 Test Technical Service Center, DeRucci Healthy Sleep Co., Ltd., Dongguan 523900, China; 2 Department of Radiology, The Second Naval Hospital of Southern Theater Command of PLA, Sanya 573000, China; 3 Joint International Research Laboratory of Environment and Health, Ministry of Education, Guangdong Provincial Engineering Technology Research Center of Environmental Pollution and Health Risk Assessment, Department of Occupational and Environmental Health, School of Public Health, Sun Yat-sen University, 74 Zhongshan 2nd Road, Yuexiu District, Guangzhou 510080, China; 4 Thornlea Secondary School, York Region District School Board, 8075 Bayview Avenue, Thornhill, ON L3T 4N4, Canada; 5 Sleep Medicine Department, Sanya Central Hospital (The Third People’s Hospital of Hainan Province), Sanya 572000, China

**Keywords:** PM_2.5_, OPEs, Pearl River Delta, children sleep disorder

## Abstract

Although particulate matter has been associated with sleep problems, the effects of PM_2.5_-bound organophosphate esters (OPEs) on children’s sleep remain unclear. OPEs have neurotoxic and endocrine-disrupting effects that may disrupt sleep–wake regulation during neurodevelopment, supporting biological plausibility for sleep impacts. In this study, we quantified the individual and mixture effects of PM_2.5_-bound OPEs on the sleep disorder domain. This cross-sectional study included 110,169 children aged 6–18 years from primary and secondary schools in the Pearl River Delta (PRD), China. Sleep disorders were evaluated using the validated Sleep Disturbance Scale for Children (SDSC). Elastic net and mixed effect models identified specific OPE–sleep associations, while weighted quantile sum regression evaluated mixture effects. All odds ratios indicate a change in the likelihood of sleep disorders per interquartile range (IQR) increase in OPE concentrations. The strongest individual associations were observed for TDCIPP with short sleep duration (OR = 1.56–1.61; moderate association), TEHP with short sleep duration (OR = 1.59–1.64; moderate association), and TPHP with overall sleep disorder (OR = 1.32–1.42; modest association). Combined OPE exposure was positively associated with all sleep disorder domains (ORs = 2.02–2.85; moderate-to-large associations). These results indicate that inhaling PM_2.5_-bound OPE mixtures could negatively impact children’s sleep health. This emphasizes a critical developmental period and highlights the importance of public health concerns related to emerging airborne contaminants.

## 1. Introduction

Organophosphate esters (OPEs) are widely used chemicals that became popular in the early 2000s as alternatives polybrominated diphenyl ethers [1,2,3]. These substances are primarily flame retardants, plasticizers, and additives used in food packaging, furniture, clothing, polishes, electronics, toys, personal care, textiles, and building materials [1,2,3,4]. Global OPE production increased from 0.68 million tons in 2016 to 0.9 million tons in 2019 [5]. China, as a major producer, increased output from 141,500 tons in 2014 to 294,200 tons in 2020 [6]. This rising production and integration highlight the increasing importance of understanding human exposure, especially among vulnerable groups.

Human exposure to OPEs primarily occurs through inhalation, ingestion, and dermal absorption. Indoor dust and airborne particles are major sources of this exposure [7]. Numerous biological and environmental matrices such as skin wipes [8], indoor air [9], silicone wristbands [10], silicone rubber brooches [11], floor and surface dust [1], blood and serum [12], urine [13], hair [14], and nails [15] have been used to characterize exposure. This is especially critical for children, due to increased hand-to-mouth activity and higher inhalation and metabolic rates [4,11,16,17]. Among these pathways, the inhalation of particle-bound OPEs warrants particular concern because semi-volatile OPEs distribute between gas and particle phases. The PM_2.5_-bound fraction is more persistent, less prone to oxidation, and capable of long-range transport [16,18,19]. Together, these characteristics indicate that PM_2.5_-associated OPEs may represent a distinct and relevant exposure pathway beyond general emissions.

Epidemiological studies link OPEs to respiratory issues like asthma, allergies, and depressive symptoms [13,20,21]. Tricresyl phosphate, a form of OPE, has been linked to neurological effects such as sleep disturbances. This demonstrates the neurotoxic potential of organophosphate esters [22,23,24]. New research suggests neurotoxic effects, with studies like Kang et al. (2022) showing a connection between OPE metabolites and sleep disturbances in adults [25]. However, the effects of OPE exposure on children’s sleep and neurodevelopment are largely unexplored and poorly understood. The neurotoxic mechanisms of OPEs are similar to organophosphate pesticides, primarily operating through the inhibition of acetylcholinesterase (AChE), which disrupts neuronal signaling and may increase the risk of sleep problems [26,27,28]. Sleep disorders are closely linked to the endocrine system, and hormonal imbalances can negatively impact sleep quality and sleep deprivation [29,30].

Children are especially vulnerable to environmental neurotoxicants like OPEs because their developing brains undergo rapid neuronal growth, synaptogenesis, and myelination, and as they have immature detox systems, this increases their chemical susceptibility [31,32]. Their sleep and circadian systems also mature early, and disruptions can harm cognitive, emotional, and behavioral development [33,34]. These mechanistic insights imply that OPE exposure could raise the risk of sleep disorders, especially in children.

The study explores the link between airborne PM_2.5_-bound OPEs and sleep disorders in children in China’s Pearl River Delta, a high-emission region. It addresses gaps by focusing on this inhalation pathway, offering new insights for public health measures to protect children’s sleep and neurodevelopment. In this study, we quantified the individual and mixture effects of PM_2.5_-bound OPEs on sleep disorder domains.

## 2. Methods

### 2.1. Study Participants

This study was conducted from May 2016 to May 2018 in the Pearl River Delta (PRD), China, with strong collaboration from 105 elementary and middle schools across six cities in Guangdong province, including Guangzhou (37 schools), Foshan (18), Zhongshan (18), Shenzhen (11), Zhuhai (8), and Maoming (13). The geographical locations of all PM_2.5_ sampling sites in the PRD are presented visually in Figure S1 of the Supplementary Materials. A total of 144,409 participants and their guardians were invited to share insights through a standardized questionnaire. The guardians’ responses provided valuable information on socio-demographic factors, lifestyle habits, health outcomes, living environments, and family history of asthma. We received 131,412 valid responses from participants, reflecting a 91% participation rate, which indicates strong community engagement. Unfortunately, data from Maoming city (n = 21,243) were excluded due to sample loss. The final sample included 110,169 children aged 6–18 from five PRD cities. Further participant characteristics are described elsewhere [35]. We are grateful for the approval from the Ethics Committee of Sun Yat-sen University (Approval Number: 2018057) and sincerely thank all guardians for their informed consent and support.

### 2.2. Measurement of Sleep Disorders

Over the past six months, sleep disorders in children have been assessed using the Sleep Disturbance Scale for Children (SDSC), based on reports from parents or guardians. This scale is frequently used in clinical and epidemiological studies to screen for sleep issues in school-aged children [36,37]. We utilized the validated Chinese version of the SDSC, which is culturally and contextually adapted for use in China [38]. The SDSC measures the following six areas of sleep disorders: disorder of initiating and maintaining sleep (DIMS), sleep–wake transition disorder (SWTD), sleep hyperhidrosis (SHY), sleep breathing disorder (SBD), disorder of arousal (DA), and disorder of excessive somnolence (DOES), using a 24-item, 5-point scale. Domain scores are calculated from the relevant items, resulting in a Global Sleep Disorder (GSD) that reflects overall sleep quality, which is then converted into t-scores [39]. Higher scores signify more sleep issues, and a t-score above 70 points to a sleep disorder [39]. Two additional questions assess total sleep duration and sleep latency, with short sleep defined as less than 7 h and long latency as more than 45 min, based on expert consensus [40,41,42]. To assess sleep disorders, a binary variable for total sleep disorder was created. It was defined as present (1) if a child exhibited one or more of the following nine specific sleep issues: DIMS, SBD, DA, SWTD, DOES, SHY, GSD, short sleep duration, and longer sleep latency. It was defined as absent (0) if none were present [42].

### 2.3. Measurement of Organophosphate Esters in PM_2.5_

PM_2.5_ samples were taken from elementary and middle schools within 1 km of air-monitoring stations in six Pearl River Delta (PRD) cities. Sampling took place during summer (May–July 2018) and winter (October–December 2018). All sites were outdoors without obstructions such as tall buildings or dense vegetation. The sample collection adhered to established protocols [43], with additional methodological details for this sampling technique provided in a previous study [35].

In this study, we examined 10 OPEs because they have recently been identified as novel PM_2.5_-bound OPEs with emerging environmental relevance and limited health evidence, as reported in Zeng et al. [44]. These are triethyl phosphate (TEP), tris(2-chloroethyl) phosphate (TCEP), tris(1-chloro-2-propyl) phosphate (TCIPP), tris(1,3-dichloroisopropyl) phosphate (TDCIPP), triphenyl phosphate (TPHP), 2-ethylhexyl diphenyl phosphate (EHDPHP), tris(2-butoxyethyl) phosphate (TBOEP), tris(2-isopropylphenyl) phosphate (T2IPPP), tris(2-ethylhexyl) phosphate (TEHP), and tris(3,4-dimethylphenyl) phosphate (T34DMPP). OPE concentrations were measured in both summer and winter. The annual mean OPE level was calculated as the average of these seasonal concentrations. For extraction, about one-eighth of each filter was ultrasonically extracted with acetonitrile and then centrifuged. The supernatant was concentrated under a nitrogen stream, exchanged into methanol, and filtered through a 0.22 µm membrane. Isotopically labeled internal standards (e.g., TCEP-d_12_, TDCIPP-d_15_, and TPHP-d_15_) were added to the extracts prior to analysis to correct for extraction efficiency and instrumental variability. OPEs were quantified by UPLC–MS/MS in MRM mode, as described by Liu et al. [45]. Validation included procedural blanks, with surrogate standards (TPHP-d_15_, TCEP-d_12_) used to monitor recoveries. The method detection limits (MDLs) are the average of field blanks plus 3 times the standard deviation or a signal of 10 times the noise level for non-detectable compounds in blanks [44]. Detection frequencies for all OPEs exceeded 95%, indicating their widespread presence in PM_2.5_ and environmental significance.

### 2.4. Covariates

The covariates were selected based on previous epidemiological studies related to children’s sleep health and environmental exposures [42,46]. The variables analyzed included age (years), sex (boys/girls), low birth weight (<2.5 kg), preterm delivery (<37 weeks), cesarean section (No/Yes), exclusive breastfeeding for at least 3 months (No/Yes), passive smoking exposure (No/Yes), pet ownership (No/Yes), home renovation within the last two years (No/Yes), visible household mold (No/Yes), daily physical activity exceeding one hour (Yes/No), and city included (Foshan, Guangzhou, Shenzhen, Zhonshang, and Zhuhai). Environmental factors considered were proximity to factories (no factory nearby; factory >100 m away; and within 100 m), and per capita living space (<20 m^2^, 20–30 m^2^, and >30 m^2^). Socioeconomic indicators included parental education (low, medium, and high) and household income (<10,000; 10,000–30,000; 30,001–100,000; and >100,000 RMB). All variables were collected through a structured questionnaire completed by parents at participating schools. This questionnaire covered modules on demographics, perinatal and early-life history, household environment, lifestyle, and socioeconomic status. The covariates described here follow the same definitions as reported in previous studies [35,42].

### 2.5. Statistical Analysis

Descriptive statistics summarized the characteristics of participants based on total sleep disorder. Continuous variables were presented as means with standard deviations and compared using Student’s *t*-tests, while categorical variables were shown as frequencies and percentages and compared with Pearson’s chi-squared tests. OPE concentrations in PM_2.5_ were summarized using percentiles and detection rates. Since exposures were right-skewed, they were standardized with IQR scaling, and regression effects were reported per IQR increase. Pairwise correlations among OPE concentrations were assessed with Pearson correlation coefficients and visualized via a heat map to evaluate interrelationships and collinearity. To identify OPEs linked to sleep disorders while managing collinearity among exposures, elastic net regression was used with adjustments for all covariates. This method combines LASSO and ridge penalties for variable selection and coefficient shrinkage with correlated pollutants. Separate models were fitted for total sleep disorder adjusting for covariates.

Associations between individual OPEs and sleep disorders were analyzed using mixed-effects logistic regression models, estimating odds ratios (ORs) and 95% confidence intervals (CIs) for each IQR increase in OPEs. The models were adjusted for all covariates included in the analysis, with city included as a random effect to account for spatial clustering. To assess the effects of OPE mixtures, weighted quantile sum (WQS) regression was employed. OPE levels were divided into quartiles to create a weighted index assessing the overall impact on sleep disorders. Covariates were included, and mixture-specific ORs along with the contribution weights of individual OPEs are provided in the Supplementary Materials.

Two sensitivity analyses evaluated the robustness of the findings. Sensitivity Analysis (1) used single-OPE mixed-effects models without scaled PM_2.5_-bound OPE concentrations, without IQR standardization. Sensitivity Analysis (2) used multi-OPE models including all OPEs to evaluate mutually adjusted associations. Both analyses aligned with the primary results and are presented in the Supplementary Materials. All tests were two-sided with *p* < 0.05, and analyses were performed using R software (version 4.4.3).

## 3. Results

Table 1 summarizes the participant characteristics by total sleep disorder status. Children diagnosed with sleep disorders tend to be older than those without such disorders, with average ages of 12.0 and 11.3 years, respectively. Children with sleep disorders had higher rates of preterm birth (6.6% vs. 5.0%) and low birth weight (7.1% vs. 4.1%). They were less physically active (29.9% vs. 31.8%) and more likely to have parents with at least a high school education. Children with sleep disorders also faced more exposures such as secondhand smoke (37.5% vs. 33.2%), household mold (45.9% vs. 40.0%), pets (20.2% vs. 17.8%), and living near factories. Conversely, recent home renovation was less common among them. Significant differences in housing conditions were observed, with more children with sleep disorders living in smaller households. The prevalence of sleep disorders also varied across cities in the Pearl River Delta.

Table 2 summarizes the concentrations of individual OPEs in PM_2.5_. These concentrations vary greatly, indicating significant exposure differences. Higher median and third-quartile concentrations were found for TEHP, TCEP, TBOEP, TPHP, and TCIPP, suggesting these compounds are more common in PM_2.5_-bound OPE mixtures. In contrast, T2IPPP, EHDPH, and T34DMPP had lower median values, though some samples showed high maximum levels, pointing to localized high-exposure areas. Additionally, several OPEs such as TEP, TCEP, TBOEP, and TEHP exhibited interquartile ranges and right-skewed distributions, emphasizing large spatial or temporal variations in ambient concentrations. The MDLs for the ten target OPEs ranged from 0.039 pg/m^3^ (T34DMPP) to 41.529 pg/m^3^ (TEP), showing high sensitivity, especially for aryl-substituted OPEs like T34DMPP and T2IPPP.

**Figure 1:**
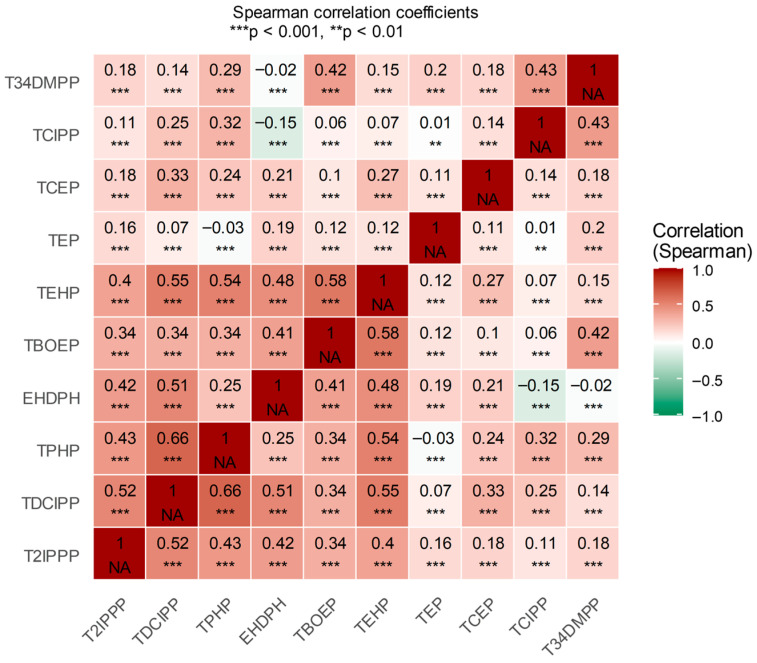
Heat map of OPEs’ network correlation in PM_2.5_.

Figure 1 shows the Pearson correlation matrix of OPEs in PM_2.5_. TDCIPP, TCIPP, TEHP, TCEP, and TEP showed strong positive correlations and formed a tight cluster, indicating co-exposure and multicollinearity. Elastic net regression was used to identify and rank OPEs linked to children’s sleep problems (Table 3) based on these correlation patterns.

Table 3 shows the elastic net regression results linking OPEs to children’s sleep problems. TDCIPP and TEHP were the most strongly associated, with high coefficients (β = 0.465 and 0.481) indicating shorter sleep duration and increased sleep latency respectively. TCIPP and TPHP also had consistent associations, while EHDPH and TBOEP had little effect. This analysis highlights the OPEs most related to adverse sleep outcomes.

Table 4 shows the overall association between organophosphate esters (OPEs) in PM_2.5_ and sleep disorders, based on generalized linear mixed models with binomial distributions that are adjusted for covariates and city-level clustering. The strongest links are for TEHP (OR: 1.22–1.61) and TDCIPP (OR: 1.24–1.59), with increased risks also seen for TCIPP (OR: 1.12–1.31) and TPHP (OR: 1.28–1.38). There are modest but significant associations for TEP (OR: 1.04–1.10), T2IPPP (OR: 1.06–1.15), TCEP (OR: 1.08–1.13), and EHDPH (1.02). TBOEP shows weak positive links (OR: 1.03–1.07) and no correlation with short sleep duration (OR: 0.99). Figure S2 visually summarizes these connections between OPEs and sleep outcomes.

Table 5 illustrates the connection between log-transformed PM_2.5_-bound OPE mixtures and pediatric sleep disorders using WQS regression. The OPE mixture was positively associated with all sleep outcomes. The strongest association is with short sleep (OR = 2.85), followed by total sleep disorder (OR = 2.74), excessive somnolence (OR = 2.54), sleep disorder (OR = 2.50), and long sleep latency (OR = 2.46). Higher odds were also found for sleep initiation and maintenance disorder (OR = 2.28), sleep breathing disorder (OR = 2.21), sleep–wake transition disorder (OR = 2.17), sleep hyperhidrosis (OR = 2.07), and arousal disorder (OR = 2.02). The contribution weights of individual OPEs to these effects vary by outcome and are detailed in Supplementary Table S1.

Table S2 in the sensitivity analysis confirms that the associations are due to OPE exposure, not to scaling artifacts. Per-unit increases in unscaled OPE analysis produced consistent, significant, and similarly ranked results. This consistency across metrics supports the robustness of the primary associations. Table S3 reveals mutual adjustment tests retain strong links for TDCIPP and TEHP, especially regarding sleep issues. Despite weaker effects from some OPEs, these two stand out, highlighting multicollinearity and indicating that high-risk compounds primarily drive population risk.

## 4. Discussion

This large-scale population study, conducted across five major cities in the Pearl River Delta (PRD), provides evidence linking ambient organophosphate esters (OPEs) in PM_2.5_ to childhood sleep disorders. We found significant associations between ten OPEs and various sleep problems. Using different analytical methods such as mixed-effects models, elastic net, and WQS regression, the study demonstrated that both individual OPEs and the mixture increased the risk of sleep disturbances, with TCEP, TCIPP, TDCIPP, TPHP, TEHP, and T34DMPP showing the strongest effects. These findings indicate that airborne OPEs, common flame retardants, could be an under-recognized environmental risk factor for childhood sleep and neurodevelopment issues.

Our findings expand on previous research linking OPE exposure to sleep disorders in adults [25], by showing similar or even stronger associations during childhood, a critical developmental period. Kang et al. (2022) [25] analyzed NHANES data to explore the relationships between urinary OPE metabolites and sleep issues in adults, identifying a positive association, mixture effects, and sex differences. Compared to Kang et al. (2022) [25], our research extends the evidence by concentrating on children and adolescents, a potentially more vulnerable group. We measured ambient exposure to 10 PM_2.5_-bound OPEs through school-based environmental sampling, targeting source-specific inhalation pathways rather than relying on internal biomarkers. Sleep was assessed with the validated Sleep Disturbance Scale for Children, enabling us to examine specific sleep disorders rather than general self-reported sleep outcomes. These features collectively offer more detailed, environmentally relevant insights into how real-world OPE mixtures impact sleep health in children. Children are a particularly vulnerable group due to their greater susceptibility to airborne pollutants, which may be attributed to higher inhalation rates per body weight, increased hand-to-mouth activity, and developing metabolic and neurological systems [11,17].

Several biological mechanisms support our findings. First, OPEs are known to disrupt thyroid endocrine regulation, affecting TSH and thyroid hormone balance [47,48,49]. Thyroid hormones regulate circadian rhythms, sleep duration, latency, and respiratory control [29,30]. Studies associate thyroid dysfunction with sleep apnea, daytime sleepiness, fragmented sleep, and delayed sleep [50,51,52,53].

Second, the neurotoxic effects of OPEs, similar to those of organophosphate pesticides, may contribute to sleep disturbances. Research has demonstrated that OPEs can affect cholinergic neurotransmission and potentially inhibit acetylcholinesterase activity [26,27,28,54,55]. Since cholinergic pathways are crucial for regulating sleep–wake cycles, these neurotoxic effects align with our observed increased risks of sleep initiation and maintenance disorders, sleep–wake transition issues, and overall sleep disturbance.

Third, OPE exposure has been associated with metabolic issues and obesity in adolescents [56,57], both of which are known risk factors for sleep problems such as snoring, disrupted sleep, and daytime fatigue [58,59]. While our study did not assess metabolic biomarkers, these pathways suggest that future research could explore how metabolic changes mediate OPEs’ impact on sleep.

In addition, tricresyl phosphate (TCP) inhibits neuropathy target esterase (NTE), linked to organophosphate-induced delayed neuropathy and neurobehavioral issues [60,61,62]. NTE, found in neurons, is involved in phospholipid metabolism vital for neuronal function [63]. Since cholinergic and neuronal phospholipid pathways affect sleep–wake behavior [64], NTE inhibition might contribute to TCP-related sleep problems, though direct evidence is limited. NTE neurotoxicity is a potential pathway needing further research.

Importantly, our analyses indicated that exposure to combined OPEs increased the risk of sleep disorders, underscoring the need to consider chemical mixtures rather than individual compounds. Similar effects of combined OPE metabolites, associated with sleep issues, appeared sex-dependent in adults using Bayesian kernel machine regression [25]. Since children are exposed to multiple OPEs through the inhalation of PM_2.5_, dust, and products [1,16], evaluating mixture effects is essential for environmental health risk assessment.

Sleep is essential for children’s neurodevelopment, cognition, and emotional regulation. Chronic disruptions can lead to long-term academic and mental health issues [65,66,67]. Our findings indicate that ambient organophosphate esters (OPEs) may play a significant role in sleep issues in children, highlighting the importance of increased public health efforts and the regulation of these pollutants.

Our study has several limitations. First, the cross-sectional design prevents drawing causal conclusions, and OPE exposure was estimated using ambient PM_2.5_ levels, which can lead to misclassification, especially where indoor pollution is significant. Second, sleep outcomes depended on parent reports rather than clinical assessments, which might have introduced bias. Third, unmeasured confounders such as diet, screen time, noise, ventilation, or psychosocial factors might influence the results. Fourth, exposure estimates were based on two seasonal samples, which may not fully capture long-term or cumulative exposure, though seasonal averages may serve as reasonable proxies. Fifth, some city–compound groups had small sample sizes, potentially inflating effects. Despite these limitations, the study provides important evidence on the health risks of ambient OPEs and highlights the need for regional actions.

## 5. Conclusions

PM_2.5_-bound OPEs are an emerging concern for children’s sleep health. This study provides epidemiological evidence that PM_2.5_-bound OPEs are linked to childhood sleep disturbances in the PRD. Both individual and combined compounds were positively associated with sleep disorders in children, with TEHP, TDCIPP, TCIPP, and TPHP identified as the most significant contributors. These positive links suggest that OPEs may harm children’s sleep and neurodevelopment. Despite limitations such as its cross-sectional design, reliance on parent reports, and potential exposure misclassification, this study addresses an important knowledge gap by identifying OPEs as an emerging environmental threat. The findings highlight the need for stricter regulations on OPE emissions and increased monitoring of exposure levels. Additionally, targeted actions in high-risk areas, such as controlling sources and improving indoor air quality in schools, are needed to protect children’s sleep and overall health.

## Figures and Tables

**Table 1:** Characteristics of participants.

Variables	Total Sleep Disorder (Yes) (n = 36,824)	Total Sleep Disorder (No) (n = 73,345)	Total Population (n = 110,169)	*p*-Value
Age, mean (SD), years	12.0 (2.9)	11.3 (2.8)	11.6 (2.8)	<0.01
Sex				0.370
Boys, n (%)	19,543 (53.1)	39,136 (53.4)	58,679 (53.3)	
Girls, n (%)	17,281 (46.9)	34,209 (46.6)	51,490 (46.7)	
Preterm birth, n (%)	2415 (6.6)	3677 (5.0)	6092 (5.5)	<0.01
Breastfeeding, n (%)	21,450 (58.3)	42,500 (57.9)	63,950 (58.0)	0.337
Physical activity, n (%)	10,791 (29.9)	23,347 (31.8)	34,138 (31.0)	<0.01
Parental education ≥ high school, n (%)	17,437 (47.4)	33,504 (45.7)	50,941 (46.2)	<0.01
Annual household income, n (%), Yuan				0.073
≤10,000	2937 (8.0)	6159 (8.4)	9096 (8.3)	
10,001–30,000	4107 (11.2)	8281 (11.3)	12,388 (11.2)	
30,001–100,000	5859 (15.9)	11,807 (16.1)	17,666 (16.0)	
100,001–200,000	12,121 (32.9)	23,775 (32.4)	35,896 (32.6)	
>200,000	11,800 (32.0)	23,323 (31.8)	35,123(31.9)	
Secondhand smoke exposure, n (%)	13,795 (37.5)	24,360 (33.2)	38,155 (34.6)	<0.01
Home renovation exposure in past 2 years, n (%)	17,170 (46.6)	37,183 (50.7)	54,355 (49.3)	<0.01
Pets at home, n (%)	7423 (20.2)	13,091 (17.8)	20,514 (18.6)	<0.01
Factory location, n (%)				<0.01
No factory	27,302 (74.1)	56,928 (77.6)	84,230 (76.5)	
Nearby	6634 (18.0)	11,785 (16.1)	18,419(16.7)	
Far away	2888 (7.8)	4632 (6.3)	7520 (6.8)	
Low Birth Weight, n (%)	2619(7.1)	3026 (4.1)	5645 (5.1)	<0.01
Caesarian, n (%)	13,998 (38.0)	27,659 (37.7)	41,657 (37.8)	0.332
Per Capita Living Area, n (%)				<0.01
Small	13,645 (37.1)	26,996 (36.8)	40,641 (36.9)	
Medium	14,758 (40.1)	31,658 (43.2)	46,416 (42.1)	
High	8421 (22.9)	14,691 (20.0)	23,112 (21.0)	
Mold Exposure, n (%)	16,903 (45.9)	29,324 (40.0)	46,227 (42.0)	<0.01
City, n (%)				<0.01
Foshan	6006 (16.3)	13,500 (18.4)	19,506 (17.7)	
Guangzhou	13,997 (38.0)	22,309 (30.4)	36,306 (33.0)	
Shenzhen	7495 (20.4)	12,223 (16.7)	19,718 (17.9)	
Zhonshang	6665 (18.1)	17,972 (24.5)	24,637 (22.4)	
Zhuhai	2661 (7.2)	7341 (10.0)	10,002 (9.1)	

Abbreviations: SD, standard deviation; *p* values are calculated for categorical variables using Pearson’s chi-squared test.

**Table 2:** The concentrations of OPEs in PM_2.5_.

OPEs	Min (pg/m^3^)	Q1 (pg/m^3^)	Median (pg/m^3^)	Q3 (pg/m^3^)	Max (pg/m^3^)	MDLs (pg/m^3^)
TEP	12.302	59.949	104.498	195.435	4531.727	41.529
TCEP	0.122	209.281	362.057	569.729	5140.226	10.991
TCIPP	18.868	80.818	111.915	172.158	747.656	10.914
TDCIPP	7.944	35.914	57.237	96.068	384.575	1.490
TPHP	0.003	122.303	323.737	573.984	2473.357	3.653
EHDPH	1.393	20.713	34.662	66.828	1379.108	5.148
TBOEP	6.738	202.701	303.260	447.972	5592.327	1.218
T2IPPP	0.474	5.954	10.378	20.876	237.297	0.219
TEHP	9.510	230.330	364.495	548.267	2415.413	1.817
T34DMPP	0.007	0.010	0.015	10.714	47.464	0.039

Abbreviations: Min, Minimum; Q1, 25th percentile; Q3, 75th percentile; Max, maximum; MDLs, method detection limits; OPEs, organophosphate Esters; TEP, triethyl phosphate; TCEP, tris(2-chloroethyl) phosphate; TCIPP, tris(1-chloro-2-propyl) phosphate; TDCIPP, tris(1,3-dichloroisopropyl) phosphate; TPHP, triphenyl phosphate; EHDPH, 2-ethylhexyl diphenyl phosphate; TBOEP, tris(2-butoxyethyl) phosphate; T2IPPP, tris(2-isopropylphenyl) phosphate; TEHP, tris(2-ethylhexyl) phosphate; T34DMPP, tris(3,4-dimethylphenyl) phosphate.

**Table 3:** Identification of OPEs associated with children’s sleep problems based on elastic network model ^a^.

β	Total Sleep Problem	GSD	DIMS	SBD	DA	SHY	DOES	SWTD	Shorter Sleep Duration	Longer Sleep Latency
TEP	0.086	0.048	0.051	0.040	0.044	0.049	0.057	0.047	0.097	0.060
TCEP	0.127	0.102	0.107	0.076	0.114	0.087	0.103	0.101	0.113	0.110
TCIPP	0.272	0.245	0.215	0.230	0.214	0.186	0.262	0.219	0.230	0.193
TDCIPP	0.410	0.275	0.277	0.238	0.233	0.243	0.295	0.217	0.465	0.382
TPHP	0.324	0.321	0.292	0.302	0.248	0.257	0.318	0.294	0.307	0.268
EHDPH	0.022	0.029	0.022	0.023	0.002	0.024	0.036	0.024	0.025	0.012
TBOEP	0.035	0.050	0.046	0.063	0.040	0.061	0.035	0.070	−0.002	0.028
T2IPPP	0.116	0.097	0.092	0.083	0.079	0.070	0.122	0.067	0.147	0.109
TEHP	0.421	0.281	0.282	0.279	0.258	0.203	0.307	0.217	0.481	0.389
T34DMPP	0.213	0.181	0.158	0.203	0.145	0.198	0.209	0.219	0.202	0.134

Abbreviations: GSD, global sleep disorder; DIMS, disorder of initiating and maintaining sleep; SBD, sleep breathing disorder; DA, disorder of arousal; SWTD, sleep–wake transition disorder; DOES, disorder of excessive somnolence; SHY, sleep hyperhidrosis. ^a^ The model was adjusted for city, age, sex, parental education level, annual household income, physical activity, secondhand smoke exposure, home renovation exposure in past 2 years, pets at home, live near factory, premature birth, breastfeeding, low birth weight, caesarean, per capita living area, and mold exposure.

**Table 4:** Association between organophosphate esters (OPE) in PM_2.5_ and odds of sleep disorders ^ab^.

Sleep Disorders	Organophosphate Esters (OPE) in PM_2.5_									
	TEP	TCEP	TCIPP	TDCIPP	TPHP	EHDPH	TBOEP	T2IPPP	TEHP	T34DMPP
	OR (95% CI)	OR (95% CI)	OR (95% CI)	OR (95% CI)	OR (95% CI)	OR (95% CI)	OR (95% CI)	OR (95% CI)	OR (95% CI)	OR (95% CI)
Total SD	1.09 (1.08–1.09)	1.13 (1.12–1.14)	1.31 (1.29–1.33)	1.50 (1.48–1.52)	1.38 (1.35–1.40)	1.02 (1.01–1.03)	1.03 (1.02–1.04)	1.12 (1.11–1.13)	1.52 (1.50–1.54)	1.23 (1.21–1.26)
GSD	1.05 (1.03–1.06)	1.11 (1.09–1.12)	1.12 (1.10–1.14)	1.31 (1.28–1.35)	1.37 (1.32–1.42)	1.02 (1.02–1.0)	1.05 (1.03–1.06)	1.10 (1.08–1.11)	1.32 (1.28–1.35)	1.19 (1.15–1.24)
DIMS	1.05 (1.04–1.06)	1.11 (1.09–1.12)	1.23 (1.21–1.26)	1.31 (1.28–1.34)	1.33 (1.29–1.37)	1.02 (1.01–1.03)	1.04 (1.03–1.05)	1.09 (1.08–1.10)	1.32 (1.29–1.35)	1.16 (1.13–1.20)
SBD	1.04 (1.02–1.05)	1.08 (1.06–1.09)	1.25 (1.22–1.28)	1.26 (1.23–1.30)	1.34 (1.30–1.39)	1.02 (1.01–1.03)	1.06 (1.05–1.07)	1.08 (1.06–1.10)	1.31 (1.28–1.35)	1.22 (1.17–1.26)
DA	1.04 (1.03–1.05)	1.12 (1.10–1.13)	1.23 (1.20–1.27)	1.26 (1.22–1.30)	1.28 (1.22–1.33)	1.00 (0.99–1.01)	1.04 (1.02–1.05)	1.08 (1.06–1.09)	1.29 (1.25–1.33)	1.15 (1.11–1.20)
SWTD	1.04 (1.03–1.06)	1.10 (1.09–1.12)	1.24 (1.21–1.27)	1.24 (1.20–1.27)	1.33 (1.28–1.39)	1.02 (1.01–1.03)	1.07 (1.05–1.08)	1.06 (1.05–1.08)	1.23 (1.20–1.27)	1.24 (1.19–1.29)
DOES	1.05 (1.04–1.07)	1.10 (1.09–1.12)	1.29 (1.36–1.32)	1.34 (1.31–1.37)	1.37 (1.32–1.42)	1.03 (1.02–1.04)	1.03 (1.02–1.04)	1.12 (1.11–1.14)	1.35 (1.32–1.39)	1.23 (1.18–1.27)
SHY	1.05 (1.04–1.06)	1.09 (1.07–1.10)	1.20 (1.17–1.22)	1.27 (1.24–1.30)	1.28 (1.24–1.33)	1.02 (1.01–1.03)	1.06 (1.05–1.07)	1.07 (1.05–1.08)	1.22 (1.19–1.25)	1.21 (1.17–1.25)
SSD	1.10 (1.09–1.11)	1.12 (1.10–1.13)	1.25 (1.23–1.27)	1.59 (1.56–1.61)	1.35 (1.32–1.38)	1.02 (1.01–1.03)	0.99 (0.98–1.00)	1.15 (1.14–1.16)	1.61 (1.59–1.64)	1.22 (1.19–1.25)
LSS	1.06 (1.04–1.07)	1.11 (1.09–1.13)	1.21 (1.17–1.24)	1.46 (1.42–1.50)	1.30 (1.25–1.35)	1.01 (1.00–1.02)	1.02 (1.01–1.04)	1.11 (1.09–1.13)	1.47 (1.43–1.51)	1.14 (1.10–1.19)

Abbreviations: OR, odds ratio; CI, confidence interval; TEP, triethyl phosphate; TCEP, tris(2-chloroethyl) phosphate; TCIPP, tris(1-chloro-2-propyl) phosphate; TDCIPP, tris(1,3-dichloroisopropyl) phosphate; TPHP, triphenyl phosphate; EHDPH, 2-ethylhexyl diphenyl phosphate; TBOEP, tris(2-butoxyethyl) phosphate; T2IPPP, tris (2-isopropylphenyl) phosphate; TEHP, tris-(2-ethylhexyl) phosphate; T34DMPP, tris(3,4-dimethylphenyl) phosphate; Total SD, total sleep disorder; GSD, global sleep disorder; DIMS, disorder of initiating and maintaining sleep; SBD, sleep breathing disorder; DA, disorder of arousal; SWTD, sleep–wake transition disorder; DOES, disorder of excessive somnolence; SHY, sleep hyperhidrosis; SSD, short sleep duration; LSS, long sleep latency. ^a^ Models were adjusted for children’s age, sex, parental education, household income, birth weight, preterm birth, cesarean delivery, breastfeeding history, second hand, smoke exposure, physical activity, presence of pets, mold exposure, nearby factory exposure, recent home renovation, and included city as a random effect. Each model included one organophosphate ester compound as the main exposure, expressed per unit increase in transformed concentration in PM_2.5_ compounds. ^b^ All *p*-values < 0.05.

**Table 5:** Associations between Organophosphate Ester (OPE) mixtures and sleep disorders using weighted quantile sum regression ^ab^.

Sleep Disorder Outcome	OR (95% CI)	Top Three Contributing OPEs
Total SD	2.74 (2.68–2.80)	TEHP, TEP, TCIPP
GSD	2.50 (2.38–2.63)	TCIPP, TEP, EHDPH
DIMS	2.28 (2.18–2.37)	TCIPP, TEP, EHDPH
SBD	2.21 (2.11–2.32)	T34DMPP, TCEP, EHDPH
DA	2.02 (1.91–2.12)	TEHP, TCIPP, TCEP
SWTD	2.17 (2.06–2.29)	T34DMPP, TCIPP, TEP
DOES	2.54 (2.42–2.64)	TCIPP, TCEP, TEP
SHY	2.07 (1.98–2.16)	T34DMPP, TCEP, TEP
SSD	2.85 (2.77–2.92)	TEHP, TEP, TDCIPP
LSS	2.46 (2.34–2.59)	TEHP, TEP, TCIPP

Abbreviations: OR, odds ratio; CI, confidence interval; TEP, triethyl phosphate; TCEP, tris(2-chloroethyl) phosphate; TCIPP, tris(1-chloro-2-propyl) phosphate; TDCIPP, tris(1,3-dichloroisopropyl) phosphate; EHDPH, 2-ethylhexyl diphenyl phosphate; TEHP, tris-(2-ethylhexyl) phosphate; T34DMPP, tris(3,4-dimethylphenyl) phosphate; Total SD, total sleep disorder; GSD, global sleep disorder; DIMS, disorder of initiating and maintaining sleep; SBD, sleep breathing disorder; DA, disorder of arousal; SWTD, sleep–wake transition disorder; DOES, disorder of excessive somnolence; SHY, sleep hyperhidrosis; SSD, short sleep duration; LSS, long sleep latency. ^a^ Models were adjusted for children’s age, sex, city, parental education, household income, birth weight, preterm birth, cesarean delivery, breastfeeding history, second hand, smoke exposure, physical activity, presence of pets, mold exposure, nearby factory exposure, and recent home renovation. ^b^ All *p*-values < 0.05.

## Data Availability

The data supporting the conclusions of this article will be made available by the corresponding authors upon reasonable request.

## Supplementary Materials

The following supporting information can be downloaded at: https://www.mdpi.com/article/10.3390/toxics14020134/s1, Figure S1: Distribution of airborne PM_2.5_ sampling sites across the Pearl River Delta region. Adapted from He et al. [68]. Figure S2: Association between Organophosphate esters (OPE) in PM_2.5_ and odds of sleep disorders. Table S1: Weight percentage of individual OPE in the association between OPEs mixture exposure and sleep problems in children. Table S2: Sensitivity Analysis 1: Associations Between Individual OPE and Sleep Disorders in Children Using Single-OPE Mixed-Effects Models (Without PM_2.5_ Scaling). Table S3: Sensitivity Analysis 2: Associations Between Multiple OPE and Sleep Disorders in Children Using Multi-OPE Mixed-Effects Models (Mutually Adjusted).
