# Supplementary material for: PM2.5-Bound Organophosphate Esters and Childhood Sleep Disorders: Evidence from the Pearl River Delta Study
Source: Toxics. 2026 Jan 29;14(2):134. doi: 10.3390/toxics14020134 (PMC12944893; doi:10.3390/toxics14020134)
Supplement: Supplementary file 1 [file toxics-14-00134-s001.zip › toxics-4087634-supplementary.pdf]

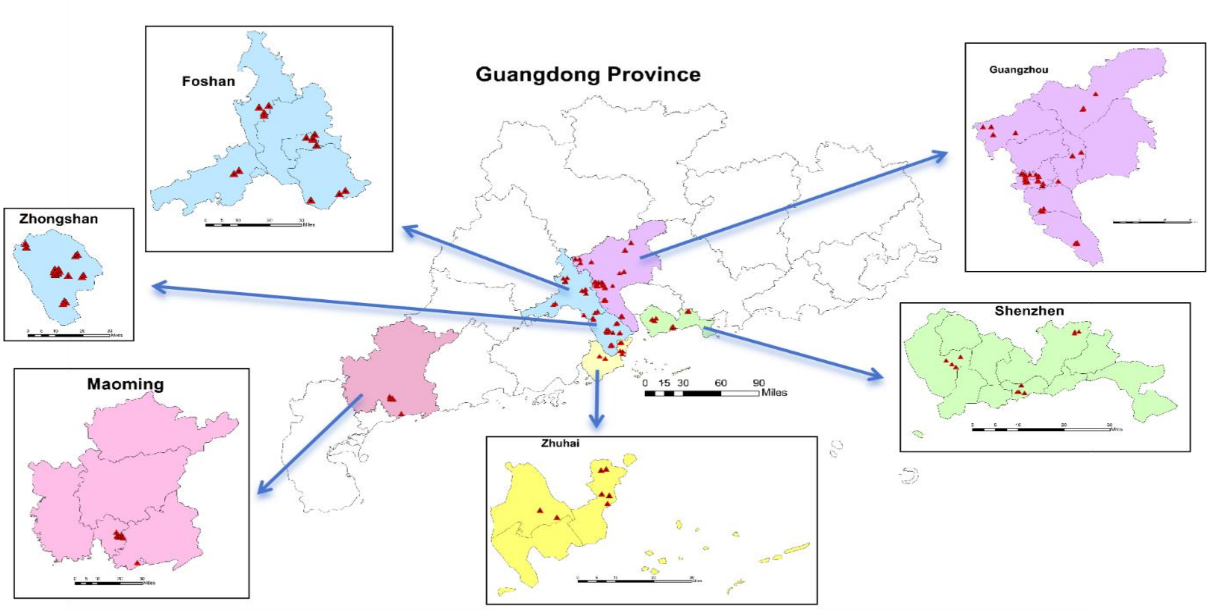

Figure S1. Distribution of airborne $PM_{2.5}$ sampling sites across the Pearl River Delta region. Adapted from He et al. [68].

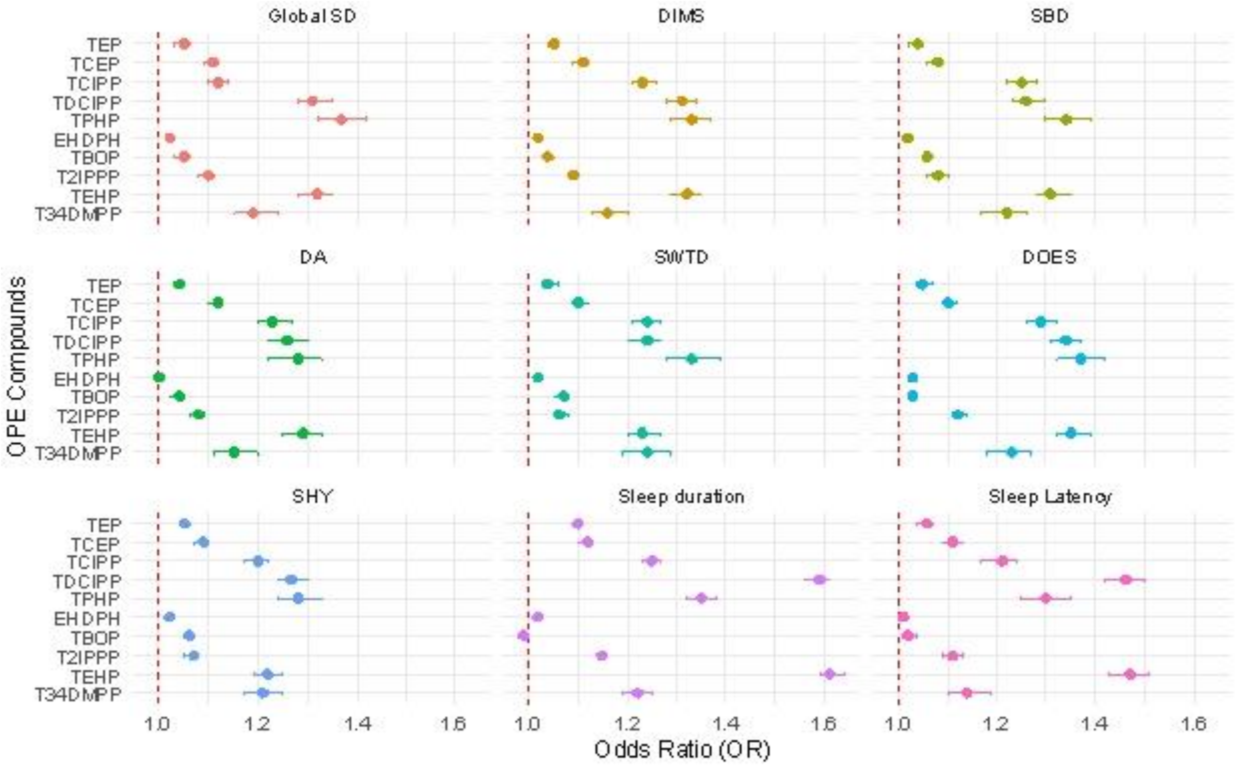

Figure S2: Association between Organophosphate esters (OPE) in $PM_{2.5}$ and odds of sleep disorders

Table S1: Weight percentage of individual OPE in the association between OPEs mixture exposure and sleep problems in children [a]

OPEs	Weight Percentage (%)									
	Total sleep problem	GSD	DIMS	SBD	DA	SHY	DOES	SWTD	Shorter sleep duration	Longer sleep latency
TEP	20.77	17.87	16.84	12.33	11.86	21.44	17.77	18.22	21.50	16.37
TCEP	15.51	16.70	15.12	18.00	18.15	20.46	19.30	16.70	9.50	8.43
TCIPP	17.95	19.30	21.13	14.63	24.25	15.03	20.57	20.35	9.68	14.45
TDCIPP	4.35	0.02	0.10	0.05	1.28	0.72	0.04	0.08	15.78	12.51
TPHP	0.02	0.00	0.00	0.01	0.06	0.03	0.00	0.01	0.00	0.01
EHDPH	14.88	17.32	17.27	18.17	10.23	11.24	14.93	15.30	11.27	13.15
TBOEP	0.07	1.60	4.36	3.80	0.74	9.81	0.02	4.94	0.00	1.33
T2IPPP	0.03	0.02	0.17	0.02	0.76	1.15	0.01	0.33	0.00	0.01
TEHP	22.50	9.98	13.72	11.32	23.61	2.78	11.1	3.22	32.27	33.71
T34DMPP	3.92	0.00	11.29	21.67	9.06	17.34	16.26	20.85	0.00	0.03

Abbreviations: GSD, global sleep disorder; DIMS, Disorder of initiating and maintaining sleep; SBD, Sleep breathing disorder; DA, Disorder of arousal; SWTD, Sleep-wake transition disorder; DOES, Disorder of excessive somnolence; SHY, Sleep hyperhidrosis; TEP, Triethyl phosphate; TCEP, Tris(2-chloroethyl) phosphate; TCIPP, Tris(1-chloro-2-propyl) phosphate; TDCIPP,Tris(1,3-dichloroisopropyl) phosphate; EHDPH,2-ethylhexyl diphenyl phosphate, TEHP,Tris-(2-ethylhexyl) phosphate; T34DMPP,Tris(3,4-dimethylphenyl) phosphate.[a] The model were adjusted for city, age, sex, parental education level, annual household income, Physical activity, secondhand smoke exposure, home renovation exposure in past 2 years, pets at home, live near factory, premature birth, breastfeeding, low birth weight, caesarian, per capita living area, and mold exposure.

Table S2: Sensitivity Analysis 1: Associations Between Individual OPE and Sleep Disorders in Children Using Single-OPE Mixed-Effects Models (Without PM 2.5 Scaling) [ab]

Sleep Disorder	Organophosphate esters (OPE) in $PM_{2.5}$									
	TEP	TCEP	TCIPP	TDCIPP	TPHP	EHDPH	TBOEP	T2IPPP	TEHP	T34DMPP
	OR(95% CI)	OR(95% CI)	OR(95% CI)	OR(95% CI)	OR(95% CI)	OR(95% CI)	OR(95% CI)	OR(95% CI)	OR(95% CI)	OR(95% CI)
Total SD	1.0006* (1.0005-1.0007)	1.0003* (1.0002-1.0004)	1.0029* (1.0028-1.0031)	1.0068* (1.0066-1.0070)	1.0007* (1.0006-1.0008)	1.0004* (1.0003-1.0005)	1.0001* (1.0000-1.0001)	1.0077* (1.0072-1.0082)	1.0013* (1.0012-1.0014)	1.0200* (1.0184-1.0217)
GSD	1.0003* (1.0003-1.0004)	1.0002* (1.0002-1.0003)	1.0026* (1.0024-1.0029)	1.0045* (1.0041-1.0050)	1.0007* (1.0006-1.0008)	1.0006* (1.0004-1.0008)	1.0002* (1.0001-1.0003)	1.0064* (1.0055-1.0073)	1.0008* (1.0007-1.0009)	1.0168* (1.0133-1.0203)
DIMS	1.0003* (1.0002-1.0004)	1.0002* (1.0001-1.0002)	1.0023* (1.0021-1.0025)	1.0046* (1.0042-1.0049)	1.0006* (1.0005-1.0007)	1.0004 (1.0003-1.0006)	1.0001* (1.0001-1.0002)	1.0061* (1.0053-1.0069)	1.0008* (1.0008-1.0009)	1.0147* (1.0116-1.0177)
SBD	1.0002* (1.0002-1.0003)	1.0002* (1.0001-1.0002)	1.0025* (1.0022-1.0027)	1.0039* (1.0034-1.0044)	1.0006* (1.0006-1.0007)	1.0005* (1.0003-1.0007)	1.0002* (1.0002-1.0003)	1.0054* (1.0045-1.0064)	1.0008* (1.0007-1.0009)	1.0189* (1.0155-1.0223)
DA	1.0003* (1.0002-1.0004)	1.0003* (1.0002-1.0004)	1.0023* (1.0020-1.0026)	1.0038* (1.0033-1.0044)	1.0005* (1.0004-1.0006)	1.0000 (0.9998-1.0002)	1.0001* (1.0001-1.0003)	1.0052* (1.0040-1.0063)	1.0008* (1.0007-1.0009)	1.0137* (1.0098-1.0176)
SWTD	1.0003* (1.0002-1.0004)	1.0003* (1.0002-1.0003)	1.0023* (1.0021-1.0026)	1.0035* (1.0030-1.0040)	1.0006* (1.0005-1.0007)	1.0005* (1.0003-1.0007)	1.0002* (1.0002-1.0003)	1.0044* (1.0034-1.0054)	1.0006* (1.0005-1.0007)	1.0204* (1.0166-1.0241)
DOES	1.0004* (1.0003-1.0005)	1.0002* (1.0002-1.0003)	1.0028* (1.0026-1.0031)	1.0049* (1.0045-1.0053)	1.0007* (1.0006-1.0008)	1.0008* (1.0006-1.0009)	1.0001* (1.0000-1.0002)	1.0081* (1.0072-1.0090)	1.0009* (1.0008-1.0010)	1.0196* (1.0162-1.0229)
SHY	1.0003* (1.0003-1.0004)	1.0002* (1.0002-1.0003)	1.0020* (1.0017-1.0022)	1.0040* (1.0036-1.0044)	1.0005* (1.0004-1.0006)	1.0005* (1.0003-1.0006)	1.0002* (1.0002-1.0003)	1.0046* (1.0037-1.0054)	1.0006* (1.0005-1.0007)	1.0184* (1.0153-1.0215)
SSD	1.0007* (1.0006-1.0008)	1.0003* (1.0003-1.0004)	1.0025* (1.0023-1.0026)	1.0077* (1.0074-1.0080)	1.0006* (1.0006-1.0007)	1.0005* (1.0004-1.0006)	0.9999 (0.9998-1.0000)	1.0099* (1.0092-1.0105)	1.0015* (1.0014-1.0016)	1.0190* (1.0170-1.0211)
LSS	1.0004* (1.0003-1.0005)	1.0003* (1.0002-1.0003)	1.0021* (1.0018-1.0026)	1.0063* (1.0059-1.0068)	1.0006* (1.0005-1.0007)	1.0002* (1.0001-1.0004)	1.0001* (1.0000-1.0001)	1.0072* (1.0061-1.0083)	1.0012* (1.0011-1.0013)	1.0126* (1.0089-1.0164)

Abbreviations: OR, Odds Ratio; CI, confidence interval; TEP, Triethyl phosphate; TCEP, Tris(2-chloroethyl) phosphate; TCIPP, Tris(1-chloro-2-propyl) phosphate; TDCIPP, Tris(1,3-dichloroisopropyl) phosphate; TPHP, Triphenyl phosphate; EHDPH,2-ethylhexyl diphenyl phosphate, TBOEP, Tris(2-butoxyethyl) phosphate; T2IPPP, Tris (2-isopropylphenyl) phosphate; TEHP, Tris-(2-ethylhexyl) phosphate; T34DMPP,Tris(3,4-dimethylphenyl) phosphate; Total SD, Total sleep disorder; GSD, global sleep disorder; DIMS, Disorder of initiating and maintaining sleep; SBD ,Sleep breathing disorder; DA, Disorder of arousal; SWTD, Sleep-wake transition disorder; DOES, Disorder of excessive somnolence; SHY, Sleep hyperhidrosis; SSD, Short sleep duration; LSS, Long sleep Latency.[a] Models were adjusted for child's age, sex, parental education, household income, birth weight, preterm birth, cesarean delivery, breastfeeding history, second hand, smoke exposure, physical activity, presence of pets, mold exposure, nearby factory exposure, and recent home renovation and city as random and city as random effect. Each model included one Organophosphate esters compound as the main exposure, expressed per unit increase in transformed concentration in $PM_{2.5}$ compounds. [b] All * indicates P- value <0.05.

Table S3: Sensitivity Analysis 2: Associations Between Multiple OPE and Sleep Disorders in Children Using Multi-OPE Mixed-Effects Models (Mutually Adjusted) [ab]

Sleep Disorder	Organophosphate esters (OPE) in $PM_{2.5}$									
	TEP	TCEP	TCIPP	TDCIPP	TPHP	EHDPH	TBOEP	T2IPPP	TEHP	T34DMPP
	OR(95% CI)	OR(95% CI)	OR(95% CI)	OR(95% CI)	OR(95% CI)	OR(95% CI)	OR(95% CI)	OR(95% CI)	OR(95% CI)	OR(95% CI)
Total SD	1.0005* (1.0004-1.0006)	1.0003* (1.0002-1.0004)	1.0013* (1.0011-1.0014)	1.0080* (1.0076-1.0081)	0.9993* (0.9992-0.9994)	0.9999 (0.9999-1.0001)	1.0000* (0.9999-1.0001)	0.9921* (0.9913-0.9928)	1.0011* (1.0010-1.0012)	1.0109* (1.0089-1.0129)
GSD	1.0003* (1.0002-1.0004)	1.0002* (1.0001-1.0003)	1.0020* (1.0017-1.0024)	1.0028* (1.0021-1.0036)	1.0000 (0.9998-1.0001)	1.0003* (1.0001-1.0005)	1.0002* (1.0001-1.0003)	0.9974* (0.9959-0.9989)	1.0005* (1.0004-1.0006)	0.9948* (0.9904-0.9993)
DIMS	1.0003* (1.0003-1.0004)	1.0002* (1.0002-1.0003)	1.0015* (1.0012-1.0017)	1.0039* (1.0032-1.0047)	0.9998* (0.9997-0.9999)	1.0001 (0.9999-1.0003)	1.0002* (1.0001-1.0003)	0.9968* (0.9955-0.9981)	1.0006* (1.0005-1.0007)	0.9984 (0.9945-1.0021)
SBD	1.0002* (1.0002-1.0003)	1.0001* (1.0000-1.0002)	1.0020* (1.0017-1.0023)	1.0001* (1.0000-1.0002)	1.0003 (1.0001-1.0005)	1.0003* (1.0002-1.0004)	1.0002* (1.0002-1.0003)	0.9976* (0.9961-0.9991)	1.0006* (1.0005-1.0007)	0.9984 (0.9941-1.0028)
DA	1.0003* (1.0002-1.0004)	1.0003* (1.0002-1.0004)	1.0016* (1.0012-1.0019)	1.0034* (1.0026-1.0043)	0.9998* (0.9996-0.9999)	0.9998 (0.9995-1.0000)	1.0002* (1.0001-1.0003)	0.9952* (0.9933-0.9970)	1.0006* (1.0005-1.0007)	0.9991 (0.9944-1.0039)
SWTD	1.0003* (1.0002-1.0004)	1.0002* (1.0001-1.0003)	1.0018* (1.0015-1.0022)	1.0020* (1.0012-1.0029)	1.0001 (0.9999-1.0002)	1.0003* (1.0001-1.0005)	1.0003* (1.0002-1.0003)	0.9975* (0.9959-0.9991)	1.0003* (1.0002-1.0005)	1.0001 (0.9954-1.0047)
DOES	1.0004* (1.0003-1.0005)	1.0002* (1.0001-1.0003)	1.0017* (1.0014-1.0020)	1.0032* (1.0026-1.0039)	0.9998 (0.9997-1.0000)	1.0004* (1.0001-1.0006)	1.0001* (1.0000-1.0002)	0.9990 (0.9976-1.0004)	1.0006* (1.0005-1.0007)	1.0033 (0.9992-1.0074)
SHY	1.0004* (1.0003-1.0005)	1.0002* (1.0001-1.0003)	1.0014* (1.0011-1.0017)	1.0034* (1.0026-1.0041)	0.9999 (0.9998-1.0000)	1.0003* (1.0001-1.0004)	1.0002* (1.0002-1.0003)	0.9965* (0.9952-0.9979)	1.0004* (1.0003-1.0005)	1.0008 (0.9968-1.0048)
SSD	1.0006* (1.0005-1.0007)	1.0003* (1.0003-1.0004)	0.9998 (0.9995-1.0000)	1.0112* (1.0108-1.0117)	0.9987* (0.9986-0.9988)	1.0002* (1.0000-1.0003)	0.9996* (0.9995-0.9997)	0.9888* (0.9879-0.9898)	1.0015* (1.0015-1.0016)	1.0254* (1.0229-1.0278)
LSS	1.0004* (1.0003-1.0005)	1.0004* (1.0003-1.0005)	1.0002 (1.0000-1.0003)	1.0089* (1.0082-1.0097)	0.9999 (0.9998-1.0000)	0.9998* (0.9996-1.0001)	0.9999 (0.9998-1.0000)	0.9898* (0.9880-0.9915)	1.0012* (1.0011-1.0013)	1.0125* (1.0079-1.0172)

Abbreviations: OR, Odds Ratio; CI, confidence interval; TEP, Triethyl phosphate; TCEP, Tris(2-chloroethyl) phosphate; TCIPP, Tris(1-chloro-2-propyl) phosphate; TDCIPP, Tris(1,3-dichloroisopropyl) phosphate; TPHP, Triphenyl phosphate; EHDPH,2-ethylhexyl diphenyl phosphate, TBOEP, Tris(2-butoxyethyl) phosphate; T2IPPP, Tris (2-isopropylphenyl) phosphate; TEHP, Tris-(2-ethylhexyl) phosphate; T34DMPP,Tris(3,4-dimethylphenyl) phosphate; Total SD, Total sleep disorder; GSD, global sleep disorder; DIMS, Disorder of initiating and maintaining sleep; SBD ,Sleep breathing disorder; DA, Disorder of arousal; SWTD, Sleep-wake transition disorder; DOES, Disorder of excessive somnolence; SHY, Sleep hyperhidrosis; SSD, Short sleep duration; LSS, Long sleep Latency.[a] Models were adjusted for child's age, sex, parental education, household income, birth weight, preterm birth, cesarean delivery, breastfeeding history, second hand, smoke exposure, physical activity, presence of pets, mold exposure, nearby factory exposure, and recent home renovation and city as random and city as random effect. Each model included one Organophosphate esters compound as the main exposure, expressed per unit increase in transformed concentration in $PM_{2.5}$ compounds. [b] * indicates P- value <0.05.
